# Emotional dampening in hypertension: Impaired recognition of implicit emotional content in auditory and cross‐modal stimuli

**DOI:** 10.1002/pchj.704

**Published:** 2023-11-09

**Authors:** Meenakshi Shukla, Rakesh Pandey

**Affiliations:** ^1^ Department of Psychology Banaras Hindu University Varanasi India; ^2^ Present address: Department of Psychology University of Allahabad Prayagraj India

**Keywords:** emotional dampening, explicit, hypertension, implicit, prehypertension

## Abstract

Research shows a reduced responsivity to implicit as well as explicit facial emotion recognition (emotional dampening) in prehypertensives and hypertensives. This study explored auditory and audiovisual emotion recognition in prehypertensives and hypertensives. Participants (*N* = 175) who were normotensives, prehypertensives, and hypertensives (*n =* 57, 58, and 60, respectively) completed an *auditory implicit task* (matching auditory target with auditory distractors) and two cross‐modal implicit tasks (matching visual target with auditory distractors, and vice‐versa), and an *auditory explicit task* (labelling emotions in audio‐clips). Findings showed an aberrant speed–accuracy trade‐off, where prehypertensives focused more on accuracy at the cost of speed while hypertensives showed the opposite. Discriminant function analysis revealed that blood pressure (BP)‐associated emotional dampening is a highly specific but moderately sensitive correlate of hypertension. Our study highlights that prehypertensives and hypertensives demonstrate emotional dampening in implicit (but not explicit) auditory emotion recognition and a greater deficit for auditory than visual recognition of implicit emotions. Findings show emotional dampening as an observable correlate of elevated BP and hypertension.

## INTRODUCTION

Hypertension is a rapidly‐increasing psychosomatic condition associated with a wide range of morbidities and mortalities and is a common risk factor for cardiovascular problems (Chobanian et al., 2003). It has been linked with emotions within the classical psychosomatic paradigm of Alexander (1939), and several studies have examined the emotion–hypertension link in terms of negative affectivity, including anxiety, depression, hostility, and anger (Jonas et al., 1997; Markovitz et al., 1991; Yan et al., 2003; Zhang et al., 2018). On one hand, such affective comorbidities common in hypertension are associated with difficulties in interpersonal relationships and reduced social support (Aslam & Alghamdi, 2017; Zhu et al., 2019). On the other, the risk of an increase in blood pressure (BP) and the development of hypertension are also predicted by reduced social support and social integration (e.g., Yang et al., 2015). This implies that affective difficulties associated with hypertension may enhance interpersonal problems in hypertensives, making adherence to treatment and the management of hypertension difficult (Krousel‐Wood & Frohlich, 2010).

Beginning in the past couple of decades, researchers have identified another affective difficulty associated with elevation in BP that may contribute to interpersonal distress, possibly leading to the development of hypertension. Termed “cardiovascular emotional dampening,” it is defined as a reduction in overall emotional responding (i.e., negative stimuli seem less negative while positive stimuli appear less positive; Pury et al., 2004) that is associated with elevation in BP (still within the normal BP range; McCubbin et al., 2011, 2014; Pury et al., 2004). Cardiovascular emotional dampening manifests in difficulties in recognizing emotions in affective faces (McCubbin et al., 2011; McCubbin et al., 2014; Shukla et al., 2018; Shukla et al., 2019) and difficulties in perceiving the emotional response of a character in a narrative (McCubbin et al., 2011, 2014). It has also been manifested as reduced valence and arousal experienced consciously while viewing positive and negative emotion‐arousing scenes from the International Affective Picture System (Pury et al., 2004) as well as a reduced unconscious experience of emotions (Shukla et al., 2020). Thus, cardiovascular emotional dampening has been defined as an “inverse relationship between resting blood pressure and emotional responsivity” (McCubbin et al., 2014), and emotion recognition difficulty has been considered as one of its indicators (McCubbin et al., 2011, 2014; Shukla et al., 2018, 2019). The present study focused on exploring the emotion recognition aspect of this BP‐associated emotional responsivity in order to develop further insight into this phenomenon.

Emotional dampening linked with elevated BP has been found to show a dose–response relationship and is evident in normotensive (McCubbin et al., 2014; Pury et al., 2004; Shukla et al., 2019) and prehypertensive (Shukla et al., 2018) individuals. The observation of dampened emotional responsiveness in elevated BP even in implicit facial emotion recognition, that is, without conscious intention to process emotions, further substantiates the likelihood that emotional dampening may be a robust correlate of elevated BP and/or hypertension. For example, Shukla and colleagues (Shukla et al., 2019) found that emotional dampening was noticeable among normotensive people in both explicit and implicit cross‐modality (visual–auditory) emotion processing, demonstrating that this phenomenon is present in both of these types of emotion processing and could be consistent across various sensory modalities (visual, auditory and cross‐modal visual–auditory). Another recently published study (Shukla et al., 2018) established for the first time that both prehypertensive and hypertensive individuals exhibit poorer recognition of dynamic facial expressions of emotions compared to people with normal BP, not only at explicit but also at implicit levels of emotion recognition. The results of this study thus offer initial evidence in favor of the idea that diminished emotional responsiveness in processing visual information is a correlate of elevated BP, regardless of whether a person has normotension, prehypertension, or hypertension.

Among the different mechanisms proposed for the development of essential hypertension, the one that is more widely implicated is the increased activity of the sympathetic nervous system, which particularly involves changes in the baroreflex pathway (Oparil et al., 2003). The baroreceptor reflex, or the baroreflex, is a bodily mechanism that acts to maintain homeostasis by keeping BP levels within a narrow range around the normal set point (Charkoudian & Rabbitts, 2009). Specialized neurons called baroreceptors located in the carotid sinus and aortic arch detect changes in BP (Charkoudian & Rabbitts, 2009). With an increase in BP, the wall of the carotid artery and the aorta stretches, causing the activation of stretch‐sensitive baroreceptors, which then convey the change in pressure to the nucleus of the tractus solitarius (NTS) located in the brainstem via the glossopharyngeal and vagus nerves (Kougias et al., 2010). The NTS is responsible for the activation and inhibition of the sympathetic and parasympathetic nervous systems (SNS and PNS, respectively). When BP increases beyond the normal set point, the NTS activates the PNS, causing the release of acetylcholine, which increases heart rate by acting on the pacemaker cells, while at the same time inhibiting the SNS and leading to a decrease in heart rate and vasodilation of blood vessels. The combined effect of these systems helps to bring BP down to normal levels.

The processes involved in BP‐induced hypoalgesia (diminished pain perception), including the opioid and baroreflex systems (Bruehl & Chung, 2004; McCubbin et al., 2006), have been proposed to contribute to emotional dampening. Active stimulation of baroreceptors has been associated with an increased threshold for pain (Rau et al., 1994). It has been speculated that emotional dampening may serve as an adaptive mechanism for keeping BP in check since perceiving emotions with full intensity (such as anger) may likely elevate BP further (McCubbin et al., 2011). On the other hand, the “baroreceptor reward hypothesis” suggests that the decrease in stress, emotional intensity, and so forth, associated with the activation of the baroreceptors may reward learned elevation in BP (Elbert et al., 1994; Mini et al., 1995), eventually leading to the development of hypertension.

Previous speculations about the mechanisms involved in BP‐associated emotional dampening state that a common central nervous system mechanism may be involved in regulating both emotional responsivity and chronic baroreflex set‐point (McCubbin et al., 2011; McCubbin et al., 2014). However, in investigating such reduced emotion recognition, previous studies (e.g., McCubbin et al., 2011, 2014; Pury et al., 2004) required the participants to label emotions or report explicitly their experience of valence and arousal from emotion‐arousing photographs, thereby assessing reduced emotional responding in the visual sensory modality alone. Thus, the available evidences are somewhat limited to conscious emotion recognition in a single modality (i.e., visual). Whether emotional responsivity in other sensory modalities and/or implicit recognition of emotions is also affected with elevation in BP has not been well‐explored. In the real world one is frequently faced with situations where emotional information is presented simultaneously through vocal (auditory) and facial (visual) expressions and understanding emotions requires parallel processing of information in both auditory and visual forms (for example, when speaking face‐to‐face with someone and simultaneously processing both facial and auditory cues of emotions). It is also important to explore whether emotional dampening is demonstrated in implicit processing of emotions. Contrary to what explicit emotion assessment measures may assume, emotions in real life are processed more automatically and do not always occur within one's awareness and may not be available to the conscious mind for verbal reporting (Weinberger et al., 1997). As a result, it is critical to examine how emotions are processed implicitly (or covertly), especially in hypertensive people who are predicted to be less emotionally responsive (e.g., McCubbin et al., 2011; McCubbin et al., 2014), using implicit (indirect) emotional assessment measures.

Thus, the present study broadly aimed at exploring the generalizability (or specificity) of cardiovascular emotional dampening by investigating whether BP‐associated emotion‐recognition deficits generalize across different sense modalities and cross‐modal processing of emotions at different levels of emotion recognition (explicit vs. implicit). In particular, the study aimed at exploring emotional dampening in auditory and visual modalities in order to extend the prior findings of emotional dampening evident in visually‐presented emotions in prehypertensives and hypertensives to auditory emotional stimuli. The study also explored how implicit recognition of emotions is affected in individuals with prehypertension and hypertension and whether it is manifested in the accuracy or speed of emotion recognition, or both. Based on findings from the prior research by Shukla et al. (2018), it was hypothesized that compared to the normotensive participants, the prehypertensive and hypertensive participants would demonstrate reduced recognition of implicit auditory and audiovisual emotional stimuli in terms of accuracy but not speed of emotion recognition. It was also hypothesized that dampening to emotions in the elevated BP groups (prehypertensive and hypertensive individuals) would be evident in both implicit and explicit tasks of emotion recognition. If sensory modality‐specific emotional dampening is observed, it would have significant implications for understanding the different brain mechanisms and areas involved in emotional dampening in hypertension. Establishing the generalizability (or specificity) of this phenomenon to a new sensory modality (auditory), and across different levels of emotional processing (implicit and explicit) may bring about a considerable increase in our knowledge of emotional dampening in hypertension.

## METHOD

### Participants

With alpha and effect size set at .05 and .30 respectively, power analysis was conducted to determine the ideal sample size for an analysis of covariance (ANCOVA), and the results showed that a sample of 175 participants was required to achieve a power of 0.95. The study included participants who met the inclusion criteria of being aged 18–60 years, and who were proficient in either Hindi or English language. Exclusion criteria encompassed individuals who were regular/daily smokers or alcohol consumers; those who were currently or previously taking medication for any other chronic physical or mental health problem; individuals with diagnosed mental disorders or a history of mental illnesses; those with physical health conditions, such as thyroid, kidney, or heart ailments; and those with vision or hearing impairments. Where a hypertension patient referred to us reported a prior history of consuming medication for their hypertension, such patients were excluded. Additionally, participants who were pregnant or breastfeeding were also excluded from the study.

Hypertensive participants were diagnosed by a trained cardiologist in the outpatient department of Sir Sunderlal Hospital, Institute of Medical Sciences, Banaras Hindu University, and the study was conducted in a silent chamber adjacent to the cardiologist's chamber. Individuals visiting the outpatient department who were diagnosed as hypertensive for the first time and were prescribed medication were immediately asked by the cardiologist to meet the researchers in the adjacent room and the newly‐diagnosed hypertensives who consented to participate were included in the study. Thus, no hypertensives were included who were on any medication for their condition. As with hypertensive participants, individuals who had their BP in the range of prehypertension and normotension were also asked by the cardiologist to contact the researchers in the adjacent room for possible participation in the research study.

A total of 175 Asian‐Indian adults (52% females and 48% males), comprising 57 normotensive (11 males and 46 females; age range = 20–41 years, mean age = 23.95 ± 3.92 years), 58 prehypertensive (31 males and 27 females; age range = 19–60 years, mean age = 32.29 ± 12.41 years), and 60 hypertensive (42 males and 18 females; age range = 20–62 years, mean age = 47.33 ± 11.93 years) participants were recruited for the study. The mean educational attainments of the normotensives, prehypertensives, and the hypertensives were 16.32 ± 2.73 years, 15.09 ± 2.81 years, and 12.87 ± 4.37 years of formal education, respectively. All the participants reported belonging to the middle‐class socioeconomic status (in accordance with the socioeconomic status classification by Khairnar et al., 2021) and reported no history of smoking, alcohol consumption, or intake of any kind of drugs. The participants described here were the same as those reported in Shukla et al. (2020). These participants were further studied on the experimental tasks reported here and the findings discussed here are novel.

In accordance with the Seventh Report of the Joint National Committee on Prevention, Detection, Evaluation, and Treatment of High Blood Pressure (JNC‐7; Chobanian et al., 2003), the inclusion criterion for normotensive individuals was systolic BP (SBP) < 120 mmHg and diastolic BP (DBP) < 80 mmHg. Prehypertensives were individuals with SBP between 120 and 139 mmHg and/or DBP in the range 80–89 mmHg. Hypertensives were individuals with SBP ≥140 mmHg and/or DBP ≥90 mmHg. The Ethics Committee, Institute of Science, Banaras Hindu University (Ref No. I.Sc./ECM‐IX/2016‐17/02) provided ethics approval for the study.

### Measures

A standard mercury sphygmomanometer was used for the BP assessment of the participants in line with the BP measurement guidelines provided by the JNC‐7 (Chobanian et al., 2003). The participants then completed four different experimental tasks: the *auditory implicit task*, the cross‐modal implicit tasks (*visual target–auditory response options task* and *auditory target–visual response options task*), and the *auditory explicit task*. Each task presented 24 trials (four for each of the six basic emotions), and each trial contained one target and four response options. Participants indicated their responses as quickly and as accurately as possible by pressing the number (1–4) corresponding to their response from the keyboard. Since, on the three implicit tasks the participants were not explicitly told to match the target and response on the basis of the similarity of emotional information presented, a correct response was considered indicative of an implicit recognition of emotions. These tasks were presented in Paradigm software (V. 2.4.0.191).

The visual and auditory stimuli used in these tasks were taken from the Cohn–Kanade AU‐Coded Facial Expression Database (Kanade et al., 2000; Lucey et al., 2010) and the IITKGP‐SEHSC (IITKGP‐SEHSC; Koolagudi et al., 2011), respectively. The Cohn–Kanade Database includes a series of monochromatic facial emotion photographs that range from the lowest to the highest intensity of emotion. This dataset is both reliable and valid, with inter‐rater agreement (kappa coefficient) of 0.75 for frame‐by‐frame coding and 0.82 for coding of the highest emotional intensity frame (Lucey et al., 2010). The auditory stimuli from IITKGP‐SEHSC contain semantically neutral Hindi statements expressed in the six basic emotional tones of happiness, sadness, fear, anger, surprise, and disgust, as well as a neutral tone. Twenty‐five postgraduate and research students from IIT Kharagpur were given a set of Hindi statements spoken in eight different tones (seven emotional and one neutral). They were then tasked with identifying the corresponding emotion from a selection of eight emotion labels. The results showed that the overall accuracy of their responses was 74% (Koolagudi et al., 2011). See Shukla et al. (2019) for details on the selection and/or development of the emotional stimuli as well as the development and validation of the three experimental tasks used in this study (except *auditory implicit task*). Therefore, only a brief description of these previously‐reported tasks has been presented below with a little more emphasis on the *auditory implicit task* (which is new).

#### Auditory implicit emotion‐recognition task

**FIGURE 1:**
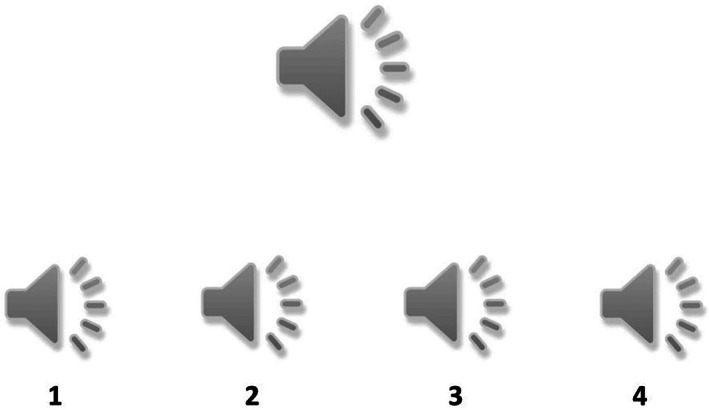
Schematic presentation of implicit auditory emotion‐recognition task.

In the auditory implicit task (Figure 1), on each trial a target audio clip with four response options consisting of audio clips labelled 1–4 appeared at the top center of the screen. The four response audio clips—numbered 1 to 4—would start playing one by one after the target audio clip had played twice. The target audio clip would automatically play twice after the fourth response audio clip had finished playing. This looping continued until the participants had entered a response. In every trial, all five audio clips delivered the same semantically neutral sentence in Hindi, such as “The sun rises in the east.” If the target audio had a male voice, each of the four response audio clips had a female voice (and vice‐versa). Participants were instructed to identify which response audio clip was the closest match to the target audio clip. Since this was an implicit task, participants were not explicitly told that the matching would entail recognition of emotions presented in the target and response audio clips. Nevertheless, since all the audio clips (target audio clip and the response audio clip options) contained the same sentence with the variation being the emotion tone of the spoken sentence, a correct matching implied an implicit/covert recognition of emotions. This task has been reported to have satisfactory reliability (Cronbach's alpha = .82) as well as concurrent and construct validities (Shukla, 2018).

#### Visual target–auditory response options cross‐modal emotion‐recognition task

**FIGURE 2:**
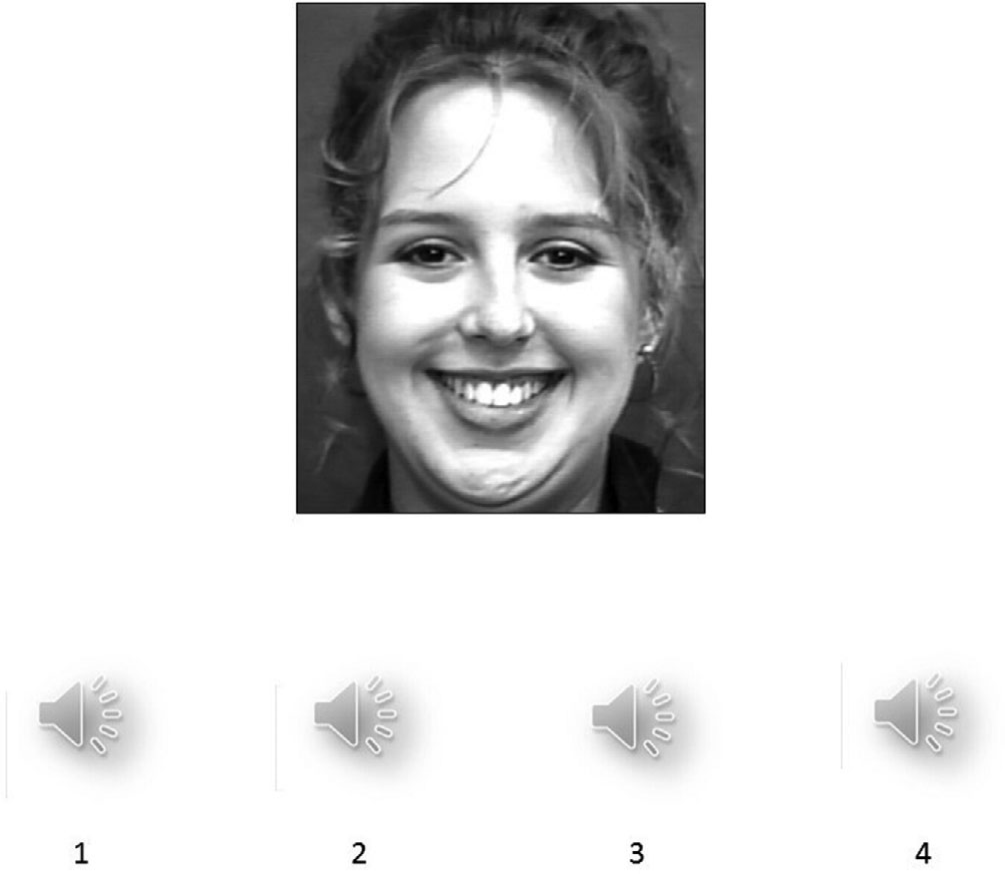
Schematic presentation of visual target–auditory response options cross‐modal task.

In the visual target–auditory response options task (Figure 2), a facial emotion video was presented as the target with four audio response options. Participants had to decide which of the response audio clips was the best match for the facial video. This was also an implicit task as the participants were not prompted explicitly to do the matching based on the similarity of emotional information. This task has satisfactory reliability (Cronbach's alpha = .71) as well as concurrent and construct validities (Shukla, 2018).

#### Auditory target–visual response options cross‐modal emotion‐recognition task

**FIGURE 3:**
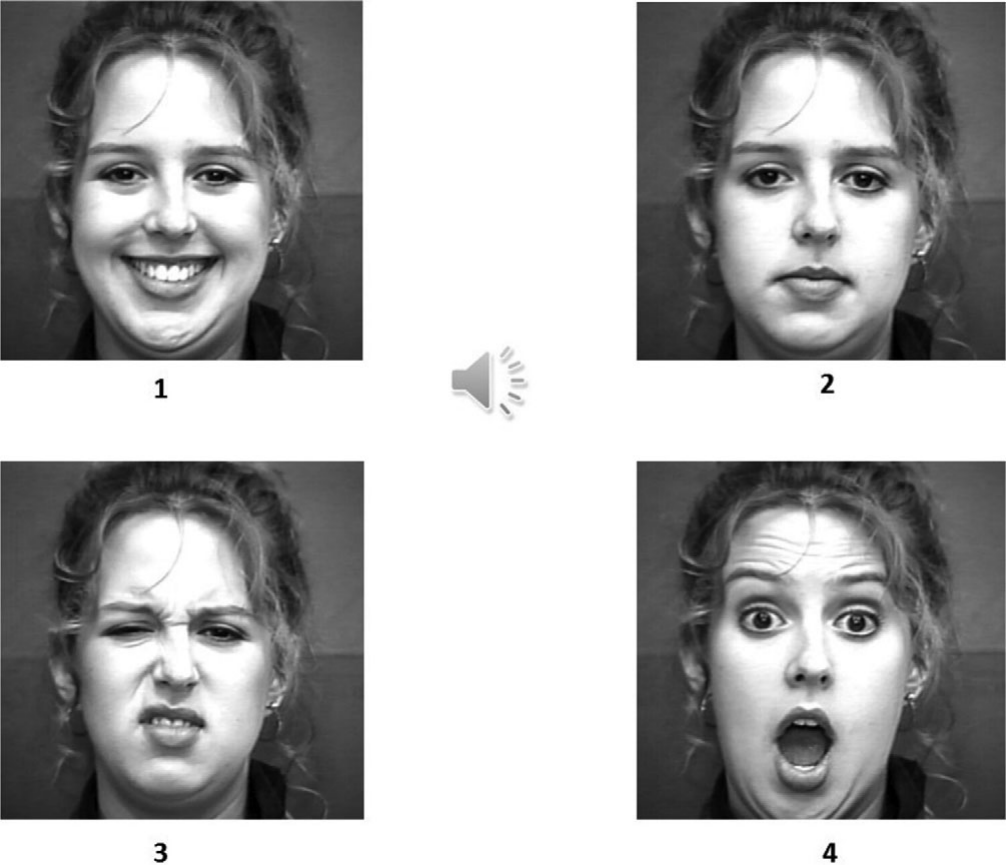
Schematic presentation of auditory target–visual response options cross‐modal task.

The auditory target–visual response options task (Figure 3) involved matching of a target audio with the equivalent facial emotion from a list of four options. The auditory clip played repeatedly until the participant had made a response. Since the participants were not explicitly instructed to match the facial emotion to the target emotion in the sound clip but asked to choose the best match for the sound clip out of the four faces displayed, correct matching indicated an implicit/covert recognition of emotions. With an internal‐consistency reliability of .74, this task has been reported to have satisfactory reliability as well as validity (both concurrent and construct; Shukla, 2018).

#### Auditory explicit emotion‐recognition task

**FIGURE 4:**
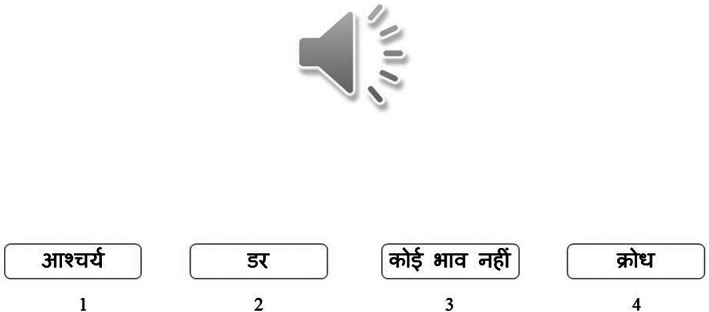
Schematic presentation of auditory explicit emotion‐recognition task.

In the auditory explicit task (Figure 4), a target audio clip was offered with four emotion labels (in Hindi). Unlike the other tasks, the participants were explicitly asked to process the emotional content of the auditory stimuli and select the matching label. One was the correct response while the remaining three options were distractors, one of which was always a “neutral” emotion label. This task has satisfactory reliability (.66) as well as validity (Shukla, 2018).

### Procedure

The study was conducted in a silent chamber adjacent to the cardiology outpatient department to ensure the availability of the cardiologist in case the participants experienced any discomfort during the experimental session. The cardiologist identified the normotensive, prehypertensive, and hypertensive individuals from those visiting the outpatient unit based on the mean score of three BP readings and the JNC‐7 criteria (Chobanian et al., 2003). There was a 2‐min gap between one BP reading and the next. Participants sat erect with feet lying flat on the ground and BP was measured from their non‐dominant arm. To ensure that the study participants' BP readings accurately reflected their status as normotensive, prehypertensive, or hypertensive people, only those participants who had not consumed caffeinated beverages, alcohol, or engaged in strenuous exercise about 2 h prior to arriving at the outpatient unit were included. These potential participants were escorted by the researcher to the experimental setting where they first provided demographic information and their written informed consent to participate in the study after receiving adequate explanations and answers to their queries about the study. During the experimental tasks, the participants sat in a comfortable height‐adjustable chair facing the computer. The viewing distance was 50 cm. For each participant, the height of the chair was adjusted in such a way so as to align the computer screen with their eyes.

Each participant completed the implicit tasks first, then the explicit tasks. This was done because an implicit task following the explicit one would have made it clear to the participants that they are to do the matching based on emotions expressed in the stimuli and would have also indicated which emotions (happiness, anger, disgust, etc.) might be present in the stimuli since the explicit task presents the emotion labels. Thus, the explicit task was presented last while the presentation of the three implicit tasks was counterbalanced across participants. Prior to each task, the participants were given task‐related instructions. After confirming that the participants had properly understood the instructions (by prompting them to repeat the instructions using their own words), they were given three practice trials. If participants had any doubt related to the task, it was resolved before administering the main experimental session. After completion of a task, a rest break of 4–5 min was provided before administration of the next task. After completion of all four tasks, the participants were debriefed.

The independent variable was BP group, with normotensives, prehypertensives, and hypertensives as its three levels, and accuracy and response time (RT) of emotion recognition were the dependent measures. The proportion of total items that were correctly answered represented the accuracy of emotion recognition. The RT data were cleaned according to the guidelines set in prior investigations (see Cepeda & Munakata, 2007; Friedman & Miyake, 2004). For every participant, the means and standard deviations of the RTs on trials with correct responses were calculated. The RTs for all the trials where participants had incorrectly identified the emotion were set to zero (zero denoted a missing value) and were excluded from further analysis. Similarly, all RT values for correct trials that were outside of the range of mean ± 3 *SD* were assigned a value of zero and designated as missing values. For each participant, the overall missing RTs from the 24 trials were computed. The median missing value for each of the three groups—normotensives, prehypertension, and hypertension—was used to determine a cut‐off for missing values for each task. The data from participants whose missing RTs exceeded the cut‐off were excluded from further analysis. If the total number of missing RTs for a participant was less than the cut‐off, the missing RT values were replaced with the mean RT of that participant. The final average RT was calculated as the mean of RTs on all the 24 trials. The number of participants for each task varied due to variations in the number of participants who were excluded from the four task conditions.

To assess the group classification accuracy of the emotion‐recognition tasks, a multiple discriminant function analysis was conducted to determine how far the accuracy of emotion recognition on the four tasks could predict the group membership of hypertensives, prehypertensives, and normotensives. Also, the receiver operating curve (ROC) analysis was conducted and sensitivity and specificity were calculated to examine the efficacy of the tasks in discriminating hypertensives from normotensives. The ROC analysis provides a comprehensive description of the accuracy of the predictors in terms of the area under the curve (AUC). According to Greiner et al. (2000), an AUC between 0.5 and 0.7 reflects that the marker is less accurate in differentiating patients from non‐patients whereas an AUC between 0.7 and 0.9 represents a moderate level of accuracy. The sensitivity of a measure refers to the proportion of individuals who have the disease (hypertensives) and give positive test results whereas specificity is the fraction of individuals that do not have the condition (normotensives) and who give negative test results. For a good marker, both the sensitivity and specificity should be high.

## RESULTS

### Accuracy of emotion recognition

With the BP groups—normotensives, prehypertensives, and hypertensives—as the independent variables and accuracy and RT of emotion recognition as the dependent measures, the results of the repeated‐measures ANCOVA (adjusting for the effects of age, gender, and education) on the four tasks (auditory implicit, visual–auditory, auditory–visual, auditory explicit) showed significant main effects of the BP groups, *F*(2, 169) = 4.904, *p* = .009, *η* ^2^ *_p_* = .055, power = 0.800, and the tasks, *F*(3, 507) = 2.720, *p* = .044, *η* ^2^ *_p_* = .016, power = 0.660, on the accuracy of emotion recognition, as well as their interaction, *F*(6, 507) = 3.759, *p* = .001, *η* ^2^ *_p_* = .043 (see Table 1). The covariates “age,” *F*(1, 169) = 85.354, *p* = .000, *η* ^2^ *_p_* = .336, power = 1.000, and “education,” *F*(1, 169) = 12.322, *p* = .001, *η* ^2^ *_p_* = .068, power = 0.937, but not “gender,” *F*(1, 169) = 0.399, *p* = .528, *η* ^2^ *_p_* = .002, power = 0.096, had significant effects on the overall accuracy of emotion recognition, such that younger and more educated participants showed significantly better recognition of emotions than older and less educated ones (bivariate correlations of education and accuracy ranged from .325 to .382 on the four tasks, *p* < .001).

**TABLE 1:** Task‐wise covariate‐adjusted mean accuracy score (in %age) of the normotensive, prehypertensive, and hypertensive groups.

Tasks	Normotensives	Prehypertensives	Hypertensives	Total Mean ± *SE*
	Mean ± *SE* [95% CI]	Mean ± *SE* [95% CI]	Mean ± *SE* [95% CI]	[95% CI]
Auditory implicit	62.43 ± 2.63	47.73 ± 2.10	46.22 ± 2.67	52.13^ab^ ± 1.20
	[57.24, 67.62]	[43.58, 51.89]	[40.94, 51.50]	[49.77, 54.49]
Visual target–auditory response options	56.17 ± 2.47	48.92 ± 1.97	44.90 ± 2.51	50.00^a^ ± 1.12
	[51.30, 61.04]	[45.02, 52.82]	[39.95, 49.86]	[47.79, 52.21]
Auditory target–visual response options	54.87 ± 2.44	53.66 ± 1.95	51.77 ± 2.48	53.43^b^ ± 1.11
	[50.05, 59.69]	[49.80, 57.51]	[46.87, 56.68]	[51.24, 55.62]
Auditory explicit	76.96 ± 2.52	76.50 ± 2.02	70.79 ± 2.57	74.75^c^ ± 1.15
	[71.98, 81.94]	[72.51, 80.48]	[65.72, 75.85]	[72.48, 77.01]
Total Mean ± *SE* [95% CI]	62.61^x^ ± 1.84	56.70^y^ ± 1.47	53.42 ^y^ ± 1.87	
	[58.99, 66.23]	[53.80, 59.60]	[49.73, 57.10]	

*Note*: a, b, c = Common superscripts (last column) denote no significant difference between means on emotion‐recognition tasks pooled across BP groups. x, y = Common superscripts (last row) denote no significant difference between means of BP groups pooled across all the four emotion‐recognition tasks.

The post‐hoc comparison of BP groups revealed that the normotensive group showed significantly higher accuracy of emotion recognition (pooled across all the four emotion‐recognition tasks) than both the prehypertensive (*p* = .003) and hypertensive (*p* = .010) groups, an indication that the individuals with elevated BPs (prehypertensives and hypertensives) made more errors in emotion recognition than the normotensives. In contrast, a post‐hoc comparison of the three BP groups across the four tasks revealed that the accuracy of emotion recognition was significantly higher in the auditory explicit task when compared to the auditory implicit task, auditory target–visual response options task, and visual target–auditory response options task (all *p*s = .000). However, the auditory target–visual response options task had a considerably higher accuracy of emotion recognition than the visual target–auditory response options task (*p* = .002), but not the auditory implicit task (*p* = .279). Similar to the visual target–auditory response options task, the accuracy of emotion recognition on the auditory implicit task did not differ substantially (*p* = .079). These findings demonstrated that participants, regardless of group membership, considered the explicit task of emotion recognition to be easier than the three implicit tasks, and during implicit cross‐modal tasks, matching an auditory target with a visual distractor was easier than matching a visual target with an auditory response option.

**FIGURE 5:**
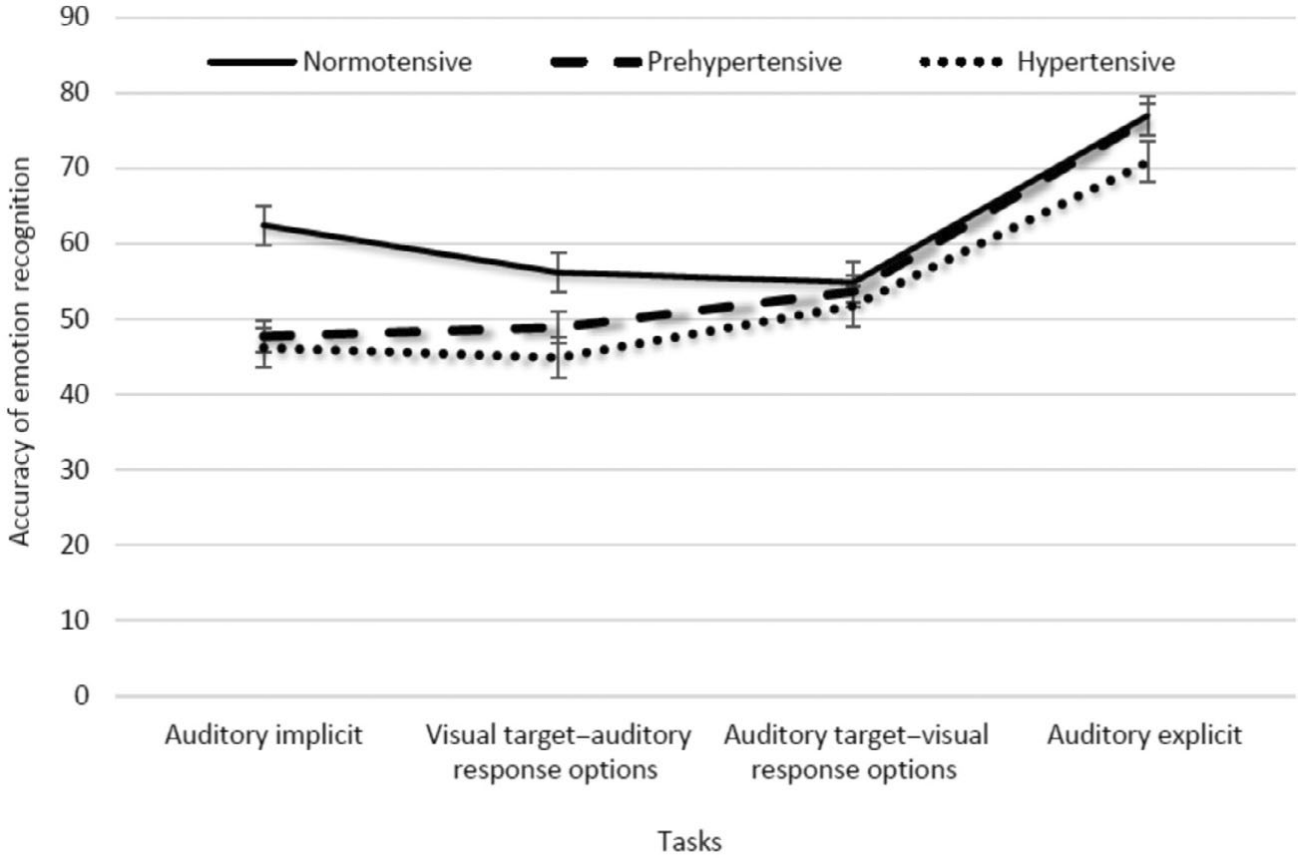
Effect of blood pressure (BP) group on the accuracy of emotion recognition across the four emotion‐recognition tasks.

It is evident, from a graphical depiction of the interaction effect (see Figure 5), that the accuracy of emotion recognition on the auditory implicit task and the visual target–auditory response options task differed among the normotensives, the hypertensives, and the prehypertensives, but not on the auditory explicit task and auditory target–visual response options task.

Notably these differences were confirmed by the results of a simple effects analysis:

1. The normotensive group showed significantly higher accuracy of emotion recognition on the auditory implicit task than on the auditory target–visual response options task (*p* < .01) in contrast to the hypertensives and prehypertensives (*p* < .01, *p* < .05, respectively), who showed significantly worse accuracy in emotion recognition.
2. The accuracy of emotion recognition on the auditory implicit task and the visual target–auditory response options task did not differ between the hypertensives and prehypertensives (mean difference = 1.314 and − 1.189, respectively; see Table 1 for means), but the normotensives showed noticeably higher accuracy on the auditory implicit task compared to the visual target–auditory response options task (mean difference = 6.258, *p* < .01).
3. The normotensives, on the other hand, demonstrated comparable levels of accuracy in emotion recognition on the two cross‐modal tasks (mean difference = 1.303, *p* = .580), while the hypertensives and prehypertensives performed significantly worse on the visual target–auditory response options task than auditory target–visual response options task (mean difference = −6.870 and − 4.734, respectively; *p* < .01 and *p* < .05, respectively).

Put together, it may be argued that the capacity of hypertensives and prehypertensives, but not normotensives, to appropriately match an implicit auditory emotional target with visual response options is somewhat insulated from the emotional dampening effects of increased BP, as evident from Figure 5.

### RT of emotion recognition

Next, we explored if the three BP groups varied in their performance in terms of their speed of recognizing emotions accurately. Separate univariate ANCOVAs with age, gender, and education as covariates were conducted to examine the effect of BP groups on average RT taken for correct responding on each of the four tasks. Group‐wise mean RTs for each task are presented in Table 2.

**TABLE 2:** Group‐wise covariate‐adjusted mean RT (in ms) of emotion recognition in the four tasks.

Blood pressure groups				
Tasks	Normotensives	Prehypertensives	Hypertensives	F_(df1,df2)_
	Mean ± *SE* [95% CI]	Mean ± *SE* [95% CI]	Mean ± *SE* [95% CI]	
Auditory implicit	25935.69^a^ ± 1062.15	29017.14^b^ ± 958.30	25773.74^a^ ± 983.59	4.06*_(2,139)_
	[23835.63, 28035.76]	[27122.40, 30911.87]	[23829.01, 27718.47]	
Visual target–auditory response options	20062.48^a^ ± 844.05	20233.00^a^ ± 782.49	18100.77^a^ ± 1148.89	1.11_(2,108)_
	[18389.43, 21735.53]	[18681.97, 21784.03]	[15823.46, 20378.07]	
Auditory target–visual response options	10014.89^ab^ ± 677.36	11678.58^a^ ± 520.94	9581.53^b^ ± 777.91	3.84*_(2,134)_
	[8675.20, 11354.58]	[10648.26, 12708.90]	[8042.97, 11120.09]	
Auditory explicit	7030.64^a^ ± 488.53	7403.36^a^ ± 415.37	6922.49^a^ ± 527.67	0.35_(2,120)_
	[6063.39, 7997.90]	[6580.94, 8225.77]	[5877.74, 7967.25]	

*Note*: a, b = Common superscripts denote no significant difference between means.

Abbreviation: RT, response time.

* *p* < .05.

Comparison of the normotensive, prehypertensive, and hypertensive groups in terms of the RT on the auditory implicit task revealed significant effect of BP on RT, *F*(2, 139) = 4.06, *p* = .019, *η* ^2^ *_p_* = .056, power = 0.718, but not on the mean RT of correctly matched cross‐modal emotions in the visual target–auditory response options condition, *F*(2, 108) = 1.11, *p* = .34, *η* ^2^ *_p_* = .02, power = 0.240. Also, there was no significant effect of BP on RT for explicit auditory task, *F*(2, 120) = 0.35, *p* = .71, *η* ^2^ *_p_* = .01, power = 0.105, suggesting that there was no difference among the three groups in their ability to quickly and accurately identify explicitly presented auditory emotions. However, a significant difference was observed across the three groups on the RT for correct cross‐modal matching (and therefore implicit identification) of auditory target stimuli with visual response options, *F*(2, 134) = 3.84, *p* = .024, *η* ^2^ *_p_* = .05, power = 0.687. Post‐hoc comparison revealed that prehypertensives took longer to respond than hypertensives (*p* = .027), albeit only a marginally significant difference in RT between the prehypertensive and normotensive groups was noted (*p* = .054; see Table 2 for means), suggesting that it may be more difficult for prehypertensives than hypertensives to cross‐modally match an auditory target with the appropriate distractor visual response. Additionally, post‐hoc analysis showed that the prehypertensives required longer RTs than the normotensives and the hypertensives (*p* = .018, *p* = .035, respectively), despite no difference between the normotensive and the hypertensive groups in their mean RTs. Because of this, the prehypertensives demonstrated delayed RT and deficit in identifying emotions, while hypertensives did not show these difficulties.

### Group classification and accuracy of the emotion‐recognition tasks

**FIGURE 6:**
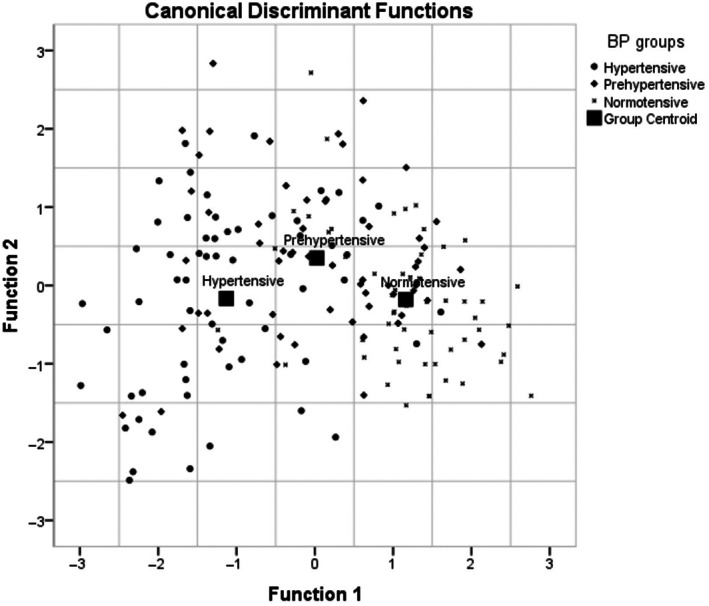
Discriminant function plot representing the group centroids of each of the three blood pressure (BP) groups against functions 1 and 2.

With accuracy of emotion recognition as a predictor and BP groups as a criterion, a multiple discriminant function analysis was performed to find evidence of emotional dampening as a manifest behavioral correlate of increased BP and hypertension. The analysis revealed two significant discriminant functions (for the first function, Wilks's lambda was 0.498, *χ* ^2^(8) = 118.86, *p* = .000, while for the second, Wilks's lambda = 0.94, *χ* ^2^(3) = 10.17, *p* = .017; see Table 3). The first function explained 93.5% of the variance, canonical correlation = .687, whereas the second function explained only 6.5% of the variance, canonical correlation = .241. Since all the tasks loaded or correlated strongly and significantly on the first function, this function was labelled as “general ability to recognize emotions,” while the second function was characterized by a single task, namely, the *auditory explicit task*, and thus was named “auditory explicit emotion recognition.” As evident from Figure 6:

1. Against function 1, which is the general ability to recognize emotions, the group centroids of the hypertensive, prehypertensive, and normotensive groups are far apart from one another, indicating that the general ability to recognize emotions strongly discriminates among the three BP groups.
2. Compared to the normotensives, the prehypertensives’ and hypertensives' group centroid is moving in the direction of the first function's origin, which suggests a gradual decline in score on this function. This may imply that, when compared to normotensives, the prehypertensives and hypertensives have shown greater dampening (or lower score) on the function of the general ability to recognize emotions.
3. Hypertensives and the normotensives were almost on the same plane, but the prehypertensive centroid was placed slightly higher than the group centroids of the normotensive and hypertensive groups, demonstrating that against the second function, which is the *auditory explicit task*, the prehypertensives can be distinguished from both hypertensives and normotensives using auditory emotion recognition, but that hypertensives and normotensives may not be distinguished by this function.

**TABLE 3:** Discriminant function structure matrix showing the correlation (loadings) of the four emotion‐recognition tasks on each discriminant function.

Tasks	Function	
	1	2
Auditory implicit	**0.872**	−0.339
Visual target–auditory response options	**0.828**	0.123
Auditory explicit	**0.671**	**0.604**
Auditory target–visual response options	**0.618**	0.376

Values in bold indicate which loading is interpreted on a given function.

The classification matrix shown in Table 4, which depicts the efficacy of each group of participants, reveals that, in terms of participant classification and efficacy, 73.3% of participants were accurately categorized as hypertensives, 37.9% as prehypertensives, and 80.7% as normotensives.

**TABLE 4:** Classification summary showing the predicted percentage of original and cross‐validated group membership of participants based on the emotion‐recognition tasks.

	Original	Predicted group membership (in %)		
	BP group	Hypertensive	Prehypertensive	Normotensive
Original^a^	Hypertensive	73.3	21.7	5.0
	Prehypertensive	25.9	37.9	36.2
	Normotensive	3.5	15.8	80.7
Cross‐validated^b^	Hypertensive	73.3	21.7	5.0
	Prehypertensive	29.3	34.5	36.2
	Normotensive	3.5	17.5	78.9

Abbreviation: BP, blood pressure.

^a^ 64.0% of originally grouped cases were correctly classified.

^b^ 62.3% of cross‐validated grouped cases correctly classified.

**FIGURE 7:**
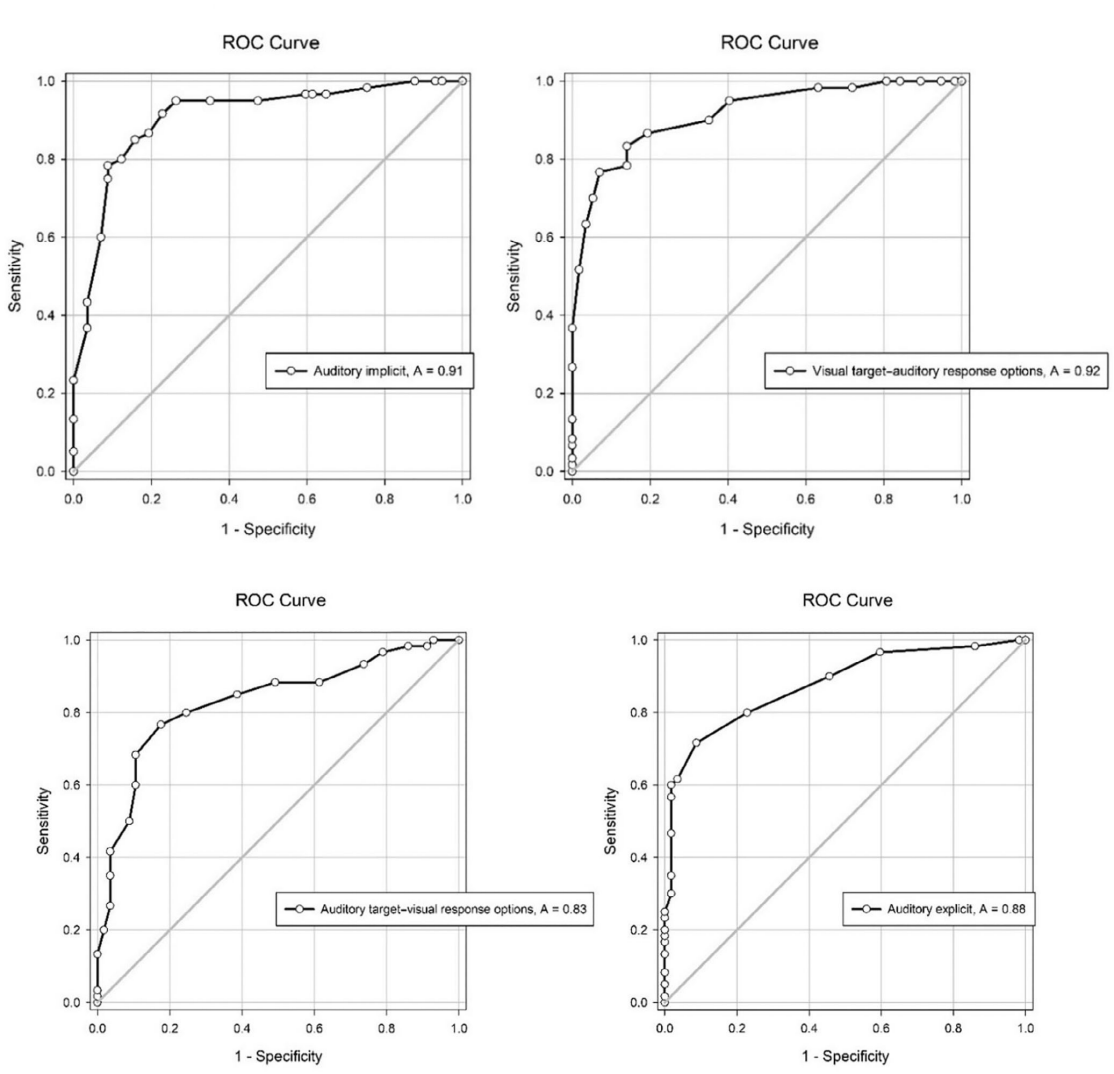
Receiver operating curve (ROC) graph showing the area under the curve (AUC) for the four indicators of hypertension.

Also, as evident from the AUC (Figure 7; the sensitivity and specificity values of which have been presented in Table 5), the accuracy of emotion recognition on visual target–auditory response options task was the highest, followed by the accuracy of emotion recognition on the auditory implicit task, auditory explicit task, and auditory target–visual response options task. The visual target–auditory response options task and auditory implicit task showed high levels of accuracy (AUC greater than 0.9). The other tasks showed moderate levels of accuracy.

**TABLE 5:** Efficacy (as indicated by AUC), sensitivity, and specificity of the behavioral emotional predictors in discriminating hypertensives from normotensives.

Emotion recognition accuracy on	AUC [95% CI]	Sensitivity	Specificity
Visual target–auditory response options task	0.916*** [0.867–0.966]	76.67%	92.98%
Auditory implicit task	0.907*** [0.851–0.963]	78.33%	91.23%
Auditory explicit task	0.878*** [0.815–0.940]	71.67%	91.23%
Auditory target–visual response options task	0.831*** [0.756–0.907]	76.67%	82.46%

Abbreviation: AUC, area under the curve.

*** *p* < .001.

## DISCUSSION

The present study attempted to explore the presence of dampening in implicit and explicit processing of emotional information presented in auditory and cross‐modality sensory conditions in individuals with hypertension and in those at risk of developing hypertension (prehypertensives). It also aimed to explore the significance of the phenomenon of emotional dampening as a measurable and observable correlate of elevated BP and hypertension. The findings of the present study revealed that the prehypertensive and hypertensive groups demonstrated poorer accuracy of emotion recognition for auditory implicit information in comparison to the normotensives. Although the prehypertensive individuals had higher accuracy of emotion recognition than the hypertensives, they did not differ significantly from the hypertensive group with respect to the accuracy of emotion recognition. Similar findings of poorer emotion recognition accuracy of both prehypertensives and hypertensives as compared to normotensives were obtained in the cross‐modal emotion recognition condition requiring the matching of correct auditory response option with visual target. The three BP groups, however, did not differ significantly with respect to the accuracy of emotion recognition on the *auditory target–visual response options* cross‐modal task or the explicit processing (labelling) of auditorily presented emotions.

With respect to the RT taken in correct unimodal implicit recognition of auditory emotions, the prehypertensives were the slowest to respond among the three groups and took significantly longer RTs for correct recognition of auditorily presented emotional information than both the normotensives and the hypertensives. In the *auditory target–visual response options task*, the prehypertensives took significantly longer in correct emotion matching as compared to the hypertensives but did not take more time than the normotensives. In the *visual target–auditory response options task* and the *auditory explicit task*, however, no difference was noted among the three groups with respect to RT. Interestingly, hypertensives and normotensives did not vary substantially with regard to the time required to accurately identify emotions. These findings provided a novel insight into the nature of emotional dampening by documenting for the first time that the speed–accuracy trade‐off (the compromise between accuracy and speed of emotion recognition) that is maintained by the normotensive individuals becomes deviant or aberrant in relation to elevated BP. However, the nature of this deviation depends on the extent of elevation in BP. In individuals with BP in the subclinical range (prehypertensives), the speed–accuracy trade‐off becomes imbalanced in such a way that these individuals focus more on accuracy at the cost of speed (prehypertensives showed better emotion‐recognition accuracy than hypertensives but slower speed of recognition compared to normotensives and hypertensives). On the other hand, if BP elevation is higher and clinically significant (in the case of hypertension), then this speed–accuracy trade‐off again shows a deviation from the normal but the deviation is characterized by a greater focus on speed at the cost of accuracy (hypertensives showed poor emotion recognition accuracy but speed was comparable to that of normotensives).

The aforementioned findings suggest that emotional dampening may be considered an observable correlate of hypertension. This speculation gets further support from the findings of the multiple discriminant function analysis: it revealed that the scores on all four tasks of emotion recognition loaded highly (*r* = .618–.872) on a single function, which accounted for more than 93% of the variance, except the scores on *auditory explicit task* that loaded equally highly on both functions 1 and 2 (*r* = .671 and .604, respectively). Function 1, named “*general ability to recognize emotions*,” discriminated each of the three BP groups from the other, while function 2, named “*auditory explicit emotion recognition*,” was important in discriminating the prehypertensive group from the normotensive and the hypertensive groups. This function did not discriminate between hypertensive and normotensive groups. Further, the two functions jointly classified 64% of the participants accurately, with 73.3% classification accuracy for hypertensives, 37.9% classification accuracy for prehypertensives, and 80.7% classification accuracy for normotensives. The cross‐validated classification accuracy of the behavioral measures was also found to be good. The emotion‐recognition tasks were also found to have high specificity and moderate sensitivity, suggesting the efficacy of these tasks in identifying emotional dampening, a behaviorally manifest correlate of elevated BP and hypertension. Here, it should be noted that the aforementioned measures may be used as tools for assessing and predicting the future risk of developing hypertension‐related emotional difficulties and not for diagnostic classification inasmuch as diagnosis of hypertension is based on well‐defined diagnostic criteria, such as that given in the seventh report of the JNC (Chobanian et al., 2003).

Overall, these findings suggest that emotional dampening is easier to assess or is more readily evident in processing of auditory than visual emotional stimuli at an implicit level of processing. This speculation is supported by the finding that the accuracy of emotion recognition was poorer in the *visual target–auditory response options task* (requiring the processing of multiple auditory stimuli) than in the *auditory target–visual response options* condition, which required processing a single auditory stimulus at a time. The present findings also extend the previous reports of emotional dampening in visual stimuli and show that emotional dampening also occurs in response to BP elevation in the prehypertensive and hypertensive ranges and that such dampening also occurs in implicit processing (recognition) of auditory emotions in uni‐ and cross‐modal conditions. Thus, emotional dampening as a consequence of high BP and hypertension may be a more fundamental phenomenon, although it manifests differentially according on the type/nature of emotional processing (e.g., implicit vs. explicit) and also varies by simultaneous processing of information from different sensory modalities. Further, the present study shows that emotional dampening may be considered a manifest correlate of elevated BP and hypertension, perhaps amenable to change through proper intervention. However, given the moderate level of sensitivity of these tasks, it may be premature to reach this conclusion and future studies are needed to further explore the sensitivity and specificity of these tasks using other similar psychosomatic conditions. Future studies may also explore other measures of assessing emotional dampening, such as the affect startle paradigm, which has recently been shown to be associated with emotional dampening at the involuntary level (Shukla et al., 2020).

This study makes significant novel additions to the literature on emotional dampening and extends the earlier findings of impaired implicit and explicit visual emotion recognition in prehypertensive and hypertensive samples (Shukla et al., 2019). The present study shows that emotional dampening associated with elevations in BP is not restricted to visually presented emotions but also extends to auditorily presented ones in prehypertensive and hypertensive populations. However, unlike the earlier studies reporting impaired implicit and explicit processing of visual emotions in prehypertensives and hypertensives (Shukla et al., 2018), the present study shows that in the auditory sensory modality, only the implicit processing of emotions is negatively affected with elevations in BP. Secondly, the study shows that BP in the prehypertensive and hypertensive ranges affects the accuracy of cross‐modal emotion processing, which requires matching of visual emotional information with auditory stimuli but not the matching of auditory emotional information with visual stimuli.

The mixed nature of findings on the cross‐modal tasks can be better understood by considering the fact that the *visual target–auditory response options task* was more similar in task parameters and requirements to the *auditory implicit task* than was the *auditory target‐ visual response options task*. Both tasks presented participants with four audio clips as response options, with the only difference being the modality of the target stimuli (visual vs. auditory, respectively). Additionally, even the findings on the said two tasks (*auditory implicit task* and *visual target–auditory response options task*) showed a similar pattern where the prehypertensive and the hypertensive groups demonstrated significantly poorer accuracy of emotion recognition compared to the normotensives but did not differ among themselves. Thus, the poor recognition of emotions in the cross‐modality condition of matching visual target stimuli with auditory response options appears to be driven by poorer recognition of auditory than visual emotional information. Further, the non‐significant difference among the three BP groups on those tasks that predominantly required decoding of auditory information (viz., *auditory explicit task* and cross‐modal emotion‐recognition task: *auditory target*—*visual response options*) coupled with the finding of an independent discriminant function solely defined by auditory task suggests the possibility that the auditory modality‐specific emotion recognition ability is relatively spared by the emotional dampening effect of elevated BP. The findings obtained on the *auditory target–visual response options task* support this conclusion as the task involved processing of only one auditory stimulus in each trial, which led to comparative performance across the three groups with respect to accuracy as opposed to the other two implicit tasks requiring processing of multiple auditory stimuli. This greater dampening to auditory than visual stimuli with elevation in BP also stands out in light of the fact that the auditory stimuli used in the present study were culturally more appropriate than the visual stimuli, and therefore should have been easier to recognize than visual stimuli, which might have posed difficulty in emotion recognition due to cross‐race effect (Elfenbein & Ambady, 2002). It should, however, be remembered that due to the very nature of the auditory stimuli, which cover a longer span of time than visual stimuli, an increase in the number of auditory stimuli to be processed may have caused a higher cognitive load resulting in poorer performance in elevated BP groups. In fact, recent research indicates a decline in cognitive functioning in elderly people with hypertension (Ihle et al., 2017). Taking such findings into account, future studies should seek to assess any deficits in working memory capacity associated with elevated BP and control for such effects in assessment of emotional dampening, particularly in prehypertensives and hypertensives.

The findings from the present study make further significant additions to the growing literature on emotional dampening. First, explicit recognition of auditory emotions appeared to be easier (based on accurate number of responses) than the implicit emotion recognition on the unimodal task. This finding is congruent with previously reported findings for explicit and implicit processing of visual emotions where greater efficiency was observed for explicit processing of emotions (Shukla et al., 2019). The present study, however, adds to earlier literature by showing that contrary to earlier findings for visual emotions, the explicit recognition of auditory emotions remains unaffected with BP elevations in the prehypertensive and hypertensive ranges. Second, our study shows that emotional dampening also occurs for implicit processing of emotions presented via the auditory sensory modality in prehypertensive and hypertensive individuals. Third, findings from the cross‐modal emotion‐recognition tasks reveal that prehypertensives and hypertensives may be more deficient in implicit processing of auditory compared to visual emotions. Fourth, an aberrant speed–accuracy trade‐off was observed with elevation in BP, such that prehypertensives focused more on accuracy at the cost of speed while hypertensives focused more on speed at the cost of accuracy.

Though the causal link between BP and emotional dampening has not yet been tested, in trying to speculate how emotional dampening may be associated with an increase in BP, some researchers (e.g., McCubbin et al., 2011, 2014) have suggested that reduced responsiveness to emotions may lead to distancing in interpersonal and social relationships due to poor emotional communication. This increasing social distance may lead to feelings of loneliness, which may cause further elevation in BP and subsequent reduction in emotional responsiveness (McCubbin et al., 2011, 2014). The finding of lower levels of emotional intelligence in hypertensive compared to normotensive individuals (Patel & Jain, 2017) also supports this speculation. A dampened response in expressing and understanding emotions may be stressful for people with prehypertension, causing repeated activation of the “fight‐or‐flight” mode and/or failure to return to normal resting levels of BP once the stressor is gone. This may lead to the development of chronically elevated BP or hypertension (McEwen, 1998). The dampened recognition of emotions expressed by others and lack of emotional sensitivity in hypertensive individuals resulting in poor interpersonal relationships may contribute to the development of negative affectivity (e.g., stress, anxiety, depression), which has already been linked with hypertension. Thus, emotional dampening may be a potential mechanism explaining the negative emotion–hypertension link and it may be a core phenomenon contributing to enhanced stress and negative affectivity observed in hypertension.

Recent studies have explored the BP–emotional‐dampening relationship with respect to some other important factors. For instance, Loveless et al. (2018) found that high resting SBP was associated with increased activity in the left frontal cortex. This enhanced frontal activity was subsequently associated with emotional dampening (neutral‐like ratings of emotional stimuli). These researchers have speculated this frontal asymmetry (an enhanced activation of the left relative to right frontal cortex) to be a possible mechanism mediating BP and emotional dampening. Since greater left compared to right frontal activation is linked to behavioral tendencies of approach (see review by Sabu et al., 2022), this implies that cardiovascular emotional dampening may be associated with enhanced behavioral approach tendencies, such as risk‐taking behaviors (e.g., rash driving, excessive drug use, and gambling; Loveless et al., 2018). Relatedly, emotional dampening has been reported to possibly mediate the relationship of high BP and increased risk‐taking behavior (McCubbin et al., 2018) due to a reduced appraisal of threat.

There are some limitations of the present study that merit consideration. First, although the BP readings used in the present study were taken during one office visit only (following the same protocol as used in earlier studies of emotional dampening), the JNC‐7 (Chobanian et al., 2003) criteria recommend that BP readings be taken on at least two or more visits. Further, the gender distribution in the present study was quite unequal and even though the effects of gender, age, and education were statistically controlled, stronger findings could have been obtained by samples matched on demographic variables. Past research has shown a cross‐race effect in emotion recognition—people typically identify emotions more swiftly and accurately in their own ethnic group than in others (Elfenbein & Ambady, 2002). Given that our study participants were ethnically distinct from the posers in the Cohn–Kanade Database (mainly Caucasians), this cross‐race effect might have influenced our results. Yet, because the images portrayed universally recognized basic emotions, any impact from this effect is likely minimal. Moreover, the tasks used had already been tested for reliability and validity on the same ethnic group (Shukla, 2018) as the participants of this study and have been reported in previous studies (e.g., Shukla et al., 2019, 2020) involving participants with the same ethnicity as this study.

## CONCLUSION

The present study provides support for the phenomenon of emotional dampening being a robust and a manifest behavioral correlate of elevated BP and hypertension. The study extends the findings of emotional dampening to unimodal processing of implicit but not explicit auditory stimuli, indicating that BP has a differential effect on the implicit and explicit processing of auditorily processed emotions. This study also extends the findings of emotional dampening in auditory stimuli to the prehypertensive and hypertensive elevations in BP. Emotional dampening due to elevated BP in the prehypertensive and hypertensive groups was also evident in the cross‐modal processing of audiovisual information; however, this effect was seen for matching of target facial emotion with its auditory counterpart, and not vice‐versa (matching audio target with visual response option). Thus, overall, it appears that implicit auditory processing is affected more than explicit processing due to elevations in BP and the difference in the cross‐modal matching of emotions is attributable more to deficits in implicit auditory than visual processing. If these findings are replicated, they would not only help expand our knowledge on the emotional dynamics associated with BP but also be helpful in identifying the mechanisms responsible for this phenomenon.

## CONFLICT OF INTEREST STATEMENT

The authors declare there are no conflicts of interest.

## ETHICS STATEMENT

The Ethics Committee, Institute of Science, Banaras Hindu University (Ref No.: I.Sc./ECM‐IX/2016‐17/02), provided ethics approval for the study.
